# Functional characterization of *Acyl-CoA Synthase 1* in tomato branch angle development

**DOI:** 10.1186/s12870-026-09516-4

**Published:** 2026-07-14

**Authors:** Mengyi Yu, Zhihuan Ge, Yinge Xie, Yongjie Qi

**Affiliations:** 1 Key Laboratory of Horticultural Crop Germplasm Innovation and Utilization (Co-Construction by Ministry and Province), Institute of Horticulture, Anhui Academy of Agricultural Sciences, Hefei, 230011 China; 2 Beijing Key Laboratory of Growth and Developmental Regulation for Protected Vegetable Crops, Department of Vegetable Science, China Agricultural University, Beijing, 100193 China; 3 Anhui Provincial Key Laboratory for Germplasm Resources Creation and High-Efficiency Cultivation of Horticultural Crops, Hefei, 230011 China; 4 Institute of Vegetables, Anhui Academy of Agricultural Sciences, Hefei, 230011 China

**Keywords:** Tomato, *SlACOS1*, Branch angle, Asymmetric growth, Auxin, Lignin

## Abstract

Branch angle is a crucial determinant of plant architecture, which is an important agronomic trait that contributes greatly to plant yield. In this study, we reported that *SlACOS1* (*Acyl-CoA synthase1*) was related to regulate the branch angle in tomato (*Solanum lycopersicum*). Knockout of the *SlACOS1* gene through CRISPR/Cas9 editing system resulted in a reduced lateral branch angle. Histological analysis revealed that editing *SlACOS1* stimulated asymmetric growth of the branch base caused by the curve growth of vascular cylinder, parenchymatous tissue incrassation, cell elongation and expansion in abaxial side (AB) of the branch. Further analyses revealed that *SlACOS1* responds to auxin and influenced its asymmetric distribution, leading to higher auxin concentration in the AB sides of the branch and subsequent differential growth of the two branch sides. In addition, *SlACOS1* interacted with two lignin biosynthesis genes *SlCCR5* and *6* to regulate lignin content in the branch. The *Slacos1* mutants bent upward more rapidly than WT in the stem gravitropism assay. However, the amyloplast sedimentation rate of statolith cells showed no significant difference between *Slacos1* and WT, suggesting *SlACOS1* functions independently of gravity. In conclusion, the *SlACOS1* contributed to asymmetric growth of branch to regulate the branch angle by modulating the asymmetric distribution of auxin and biosynthesis of lignin.

**Supplementary Information:**

The online version contains supplementary material available at https://doi.org/10.1186/s12870-026-09516-4.

## Background

Branch (or tiller) angle, defined as the angle between the main culm and its side branch, is one of the most important agricultural traits [1, 2]. Branch angle is determined by genetic and phytohormonal pathways, and also modulated by environmental factors such as light, gravity and planting density [3–6]. In *Arabidopsis*, *LAZY1* is the first identified gene that regulates the branch angle and promotes upward shoot orientations [7]. *lazy1* (*la1*) mutant exhibits wide-angled branch phenotype due to disrupted shoot gravitropism and altered auxin transport [7]. *TAC1* and *LAZY1* both belong to *IGT* gene family that regulate lateral shoot orientation in plants [8]. *TAC1* promotes outward orientations via establishing wide branch angle in response to light. Meanwhile, *TAC1* and *LAZY1* show opposing functions in regulating lateral branch angle [9]. In rice (*Oryza sativa*), *OsTAC1* is isolated by map-based cloning and participates in the regulation of rice tiller angle [10]. The *Ostac1* mutant displays a smaller tiller angle, resulting in a more compact plant architecture that contributes to rice yield substantially and is broadly used in the breeding of Asian cultivated rice [10, 11].

Phytohormones act as plant growth regulators that modulate the size of branch angle, especially auxin, brassinosteroid, cytokinin and strigolactone [3]. Asymmetrical auxin distribution within plant organs explains the mechanism of tropic growth of branch [12, 13]. Auxin transport has been thought to be a central component of branch angle signaling [7]. Loss of *HSFA2D* function enhances polar auxin transport (PAT) and impairs lateral auxin transport, resulting in tiller-spreading phenotype [12]. Suppressing of rice *PIN1* (*PIN-FORMED1*) or over-expression of rice *PIN2* (*PIN-FORMED2*), two auxin efflux transporters, both alter PAT and increase tiller angles [14]. Additionally, the maize LAZY1 protein can directly bound to the promoter of a nuclear-localized Aux/IAA auxin signaling repressor gene *ZmIAA17* to regulate lateral organ angle [15]. Strigolactone (SL) acts in regulating branch angle indirectly by suppressing auxin biosynthesis [16]. Brassinosteroid (BR) is also involved in the regulation of both tiller and leaf angle. The rice *dwarf61* (*d61*) mutant indicates the role of BR signaling: *d61* exhibits erect leaves owing to the impaired development of the lamina joint [17]. And rice *Leaf and Tiller Angle Increased Controller* (*LAX*) gene regulates tiller and leaf angle through BR signaling [18].

There is a growing body of evidence that vertically oriented branch architectures have been shown to enhance crop productivity in horticultural crops, as observed in cereal crops. For the fruit tree apple (*Malus domestica*), a trait called columnar or *Co*, which has narrow branch angles and higher apical dominance, has been bred for integration into high-density orchard systems [19]. The *Co* locus has been shown to contain a Ty3-type transposable element that is not inserted within a known gene but may act, in part, through altered expression of a neighboring 2-ODD gene [20]. Peach (*Prunus persica*) trees carrying the “Pillar” or “Broomy” trait have erectly oriented branches and narrower canopy caused by loss-of-function mutations in *TAC1* gene [21]. In rapeseed (*Brassica napus*), genome-wide association mapping (GWAS) has revealed genetic control of branch angle, with positional variation in branch angles evaluated across individual plants [22]. Tomato (*Solanum lycopersicum*) is one of the most important vegetable crops cultivated worldwide and a commonly used model species for plant biology [23]. However, little was known about the tomato lateral branch angle.

In current study, a tomato *Acyl-CoA synthase 1* gene, *SlACOS1*, was identified. *ACOS*, encoding a fatty Acyl-CoA synthetase, belongs to a supergene family *4-coumarate*: *CoA ligase* (*4CL*) [24, 25]. Our study suggested that editing *SlACOS1* using CRISPR/Cas9 system resulted in a phenotype with a smaller branch angle in tomato line PI 114,490. Histological analysis revealed that the curve branch was caused by the cell elongation and expansion on the abaxial side (AB) of the branch base. Further analysis demonstrated that the curved branch was caused by the asymmetric auxin distribution. Besides, *SlACOS1* was involved in regulating lignin content by interacting with two lignin biosynthesis genes, *SlCCR5* and *6*, supporting the branch bending. In a word, our result provided novel insights into the function of *SlACOS1* in regulating branch angle and underlying mechanisms of this physiology process.

## Methods

### Plant materials and growth conditions

Tomato (*Solanum lycopersicum* var. *cerasiforme* accession PI 114490) was used as the plant material for all experiments in this study. All plant materials were transplanted into the greenhouse 45 days after sowing with consistent water and fertilizer management.

### Generation of mutant plants

**Fig. 1:**
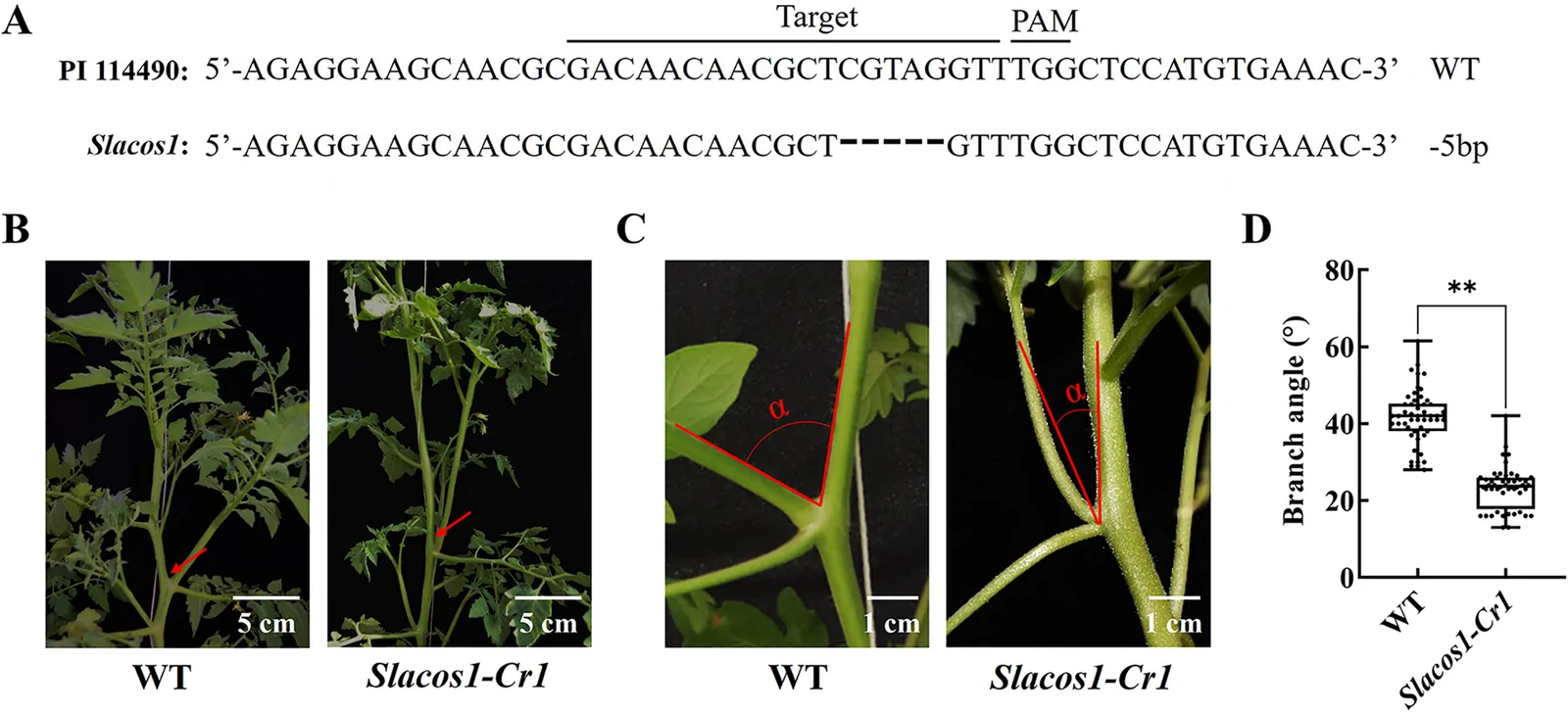
Knockout of *SlACOS1* using CRISPR/Cas9 altered the branch angle. **A** Sequences of *Slacos1* mutants generated by CRISPR/Cas9 editing. **B** Phenotype of WT (wild-type, tomato line PI 114490) and *Slacos1* mutant edited by CRISPR/Cas9; **C** Magnified images of branch of WT and *Slacos1.* The *α* indicated with red lines represented the branch angle; **D** Box-plot diagram analyzed the statistical analysis of branch angle in WT and *Slacos1.* Values are means ± SD (*n* = 60). Student’s *t*-test: **, *P* < 0.01

The CRISPR/Cas9-editing system [26] was used to generate mutants of *Slacos1* in tomato. Target sites (Single-guide RNAs, sgRNA) for the *SlACOS1* gene were designed using the released tomato reference genome (SL 2.5) from CRISPR-P 2.0 (http://crispr.hzau.edu.cn/CRISPR2/; Fig. 1A). The sgRNA fragments inserted into a recombinant CRISPR/Cas9 vector pCBC-DT1T2 with tomato U6 promotor according to the methods of the previous description [27]. The tomato genetic transformation was performed using the previous methods [28].

### Histological analysis

Longitudinal and transverse sections of lateral branches were observed via paraffin sectioning. FAA solution and 0.1% Toluidine Blue O were purchased from Beijing Solarbio Science and Technology Co. (Beijing, China). Experimental procedures were performed referring to Xie et al. [29] and reagent instructions with some modifications.

### Amyloplast sedimentation analysis

For amyloplast sedimentation analysis, a Periodic Acid Schiff Stain kit (Beijing Solarbio Science and Technology Co., Ltd) was used according to the manufacturer’s directions. Briefly, tomato plants were reoriented by 180° for 10, 20, 30 and 40 min, and lateral branches were cut into 0.5 cm fragments and fixed with FAA solution. Samples were embedded in paraffin and cut into 10 μm sections using the method described as above. Then, cross-sections were oxidized for 10 min in 0.5% periodic acid in 0.3% nitric acid, rinsed in running water for 1–2 min with a final rinse in distilled water, stained for 15 min in Schiff’s reagent, washed three times in 0.5% sodium metabisulfite for 2 min each, rinsed for 5 min in running water and then transferred to distilled water, dehydrated with graded ethanol and sealed with neutral balsam. Stained sections were observed microscopically.

### Hormones treatment and measurement

To investigate the relationship between *SlACOS1* and hormones, we tested the induction of *SlACOS1* by IAA, CTK and BR. WT seedings were treated with individual phytohormones when plants have 5 fully expanded true leaves. Three separate plants groups were sprayed to leaves of the whole plant with only one phytohormone, 20 µM IAA (Indole-3-acetic acid), 50 µM 6-BA (Cytokinin) and 50 µM BR (Brassinosteroid). After treatment for 0, 0.5, 1, 2, 4, 8, 12 and 24 h, three simple leaves at the same stem node were detached, and rapidly frozen in liquid nitrogen and stored at -80 °C until further use. The experiments were repeated three times with a total of 9 tomato plants for each treatment.

To detect the phytohormones content of lateral branch, tomato plants at the nine-leaf stage were used. The 3rd, 4th and 5th lateral branches (numbered acropetally from the first true leaf) were excised from each plant. Based on previously description [30], we defined the half of upper/lower side of the branch named adaxial side (AD)/ abaxial side (AB; Fig. S1). The lateral branches were transversely and horizontally divided into two equal parts as AD and AB samples. Tissue from three individual plants were pooled together to form one biological sample, and three independent biological replicates were prepared. The extraction and quantification of IAA, 6-BA and BR were performed using the procedure as described previously [31, 32], and the experiments were performed by the Wang Baomin’s laboratory (Center of Crop Chemical Control, China Agricultural University, Beijing, China).

### Gene transcript analysis

All the analysis of gene expression was performed by quantitative real-time polymerase chain reaction (RT-qPCR) in the current study. Total plant RNA extraction and first-strand cDNA synthesis were performed using Quick RNA Isolation Kit (Huayueyang Biotechnology Co., Beijing, China) and PrimerScript™ RT Reagent Kit with gDNA Eraser (Takara Bio Inc., Dalian, China) according to the manufacturer’s protocols. RT-qPCR was performed in 10 µl reactions containing 5 µl of SYBR Green Premix Ex Taq II (Takara Bio Inc.), 0.5 µl of 1.0 µM of each primer, 1 µl of cDNA templates and 3 µl of sterile distilled H_2_O, using the Applied Biosystems^®^ QuantStudio™ 6 Flex real time-PCR machine (Bio-Rad, California, USA). DNA amplification was conducted using the following thermocycling program: 40 cycles at 95 °C for 30 s and 60 °C for 30 s. After amplification, the samples were maintained at 55 °C for 1 min, and the temperature was gradually increased by 0.5 °C every 10 s to perform the melting curve analysis. The tomato gene *EF-1α* (*Solyc06g0050600*) was used as the internal control [33]. And reactions for the reference genes were included in each plate. Each sample had three biological and technical replicates, and the gene relative expression was calculated via 2^-△△Ct method [34]. The standard deviation was calculated from three biological replicates. RT-qPCR primers (Table S1) for each gene were designed by the National Center for Biotechnology Information (NCBI; https://www.ncbi.nlm.nih.gov/tools/primer-blast/).

### Gene interaction analysis

For yeast two-hybrid screening, *SlACOS1*-BD fragment was amplified by PCR using primers (Table S1). The *SlACOS1*-BD plasmid was used to screen the yeast library of tomato leaf in the pGADT7-AD vector. And the yeast strain AH109 was transformed via Takara Matchmaker yeast two-hybrid kit (Takara Bio Inc.) following official protocols.

For Bimolecular Fluorescence Complementation (BiFC), PCR products generated using primers *SlACOS1*-YN and *SlCCR5/6*-YC (Table S1) were connected to pSPYNE and pSPYCE vectors digested with *Bam* HI and *Kpn* I restrictions enzymes to generate *SlACOS1*-pSPYNE and *SlCCR5/6*-pSPYCE recombinant vectors, respectively. Tobacco leaf inoculation and microscopic observation were conducted following the method described by Xie et al. [29].

### Statistical analysis

Statistical analysis was carried out with GraphPad Prism 8.0 (GraphPad Software Inc., California, USA; http://www.graphpad.com/). A paired Student’s t-test was adopted for data significance testing using SPSS (https://www.ibm.com/products/spss-statistics). All results are presented as mean ± standard deviation (SD) based on no fewer than three biological replicates. Significant differences compared with the control group were marked as *p < 0.05 and **p < 0.01.

## Results

### Mutation of *SlACOS1* influences the branch angle in tomato

In order to obtain clues on the function of *SlACOS1* in the development of tomato, the transcript accumulation of *SlACOS1* gene in different tissues, including root, stem, axillary bud, young branch, mature branch, young leaf, mature leaf, flower, mature green fruit and ripening fruit were detected by quantitative RT-PCR. RT-qPCR results showed that *SlACOS1* transcripts were present in all tissues tested and abundant in stem, axillary bud and young branch (Fig. S2). The highest transcripts levels were in axillary bud, where they were 8.2-fold higher than in the reference (root) tissue (Fig. S2). The expression parents of *SlACOS1* in different tissue suggested that it maybe play roles in the branch growth.

To verify the function of *SlACOS1* in tomato, CRISPR/Cas9 editing system was applied to knock out *SlACOS1* in PI 114,490 (Fig. 1A). The phenotype of WT and *Slacos1* mutants were showed in Fig. 1B and C. The lateral branch of *Slacos1* lines were more erect and upward growth compared with WT. The angle between lateral branch and main stem was defined as the branch angle denoted by alpha (*α*, Fig. 1C). Statistical analysis of branch angle indicated that the average branch angle was 45° and 27°, respectively in WT and *Slacos1*. The *Slacos1* branch angle was significantly (*p* < 0.01) smaller than that of WT (Fig. 1D). The results revealed that *SlACOS1* gene played roles in the growth angle of lateral branch in tomato.

### *SlACOS1* regulates the asymmetric growth of the branch

**Fig. 2:**
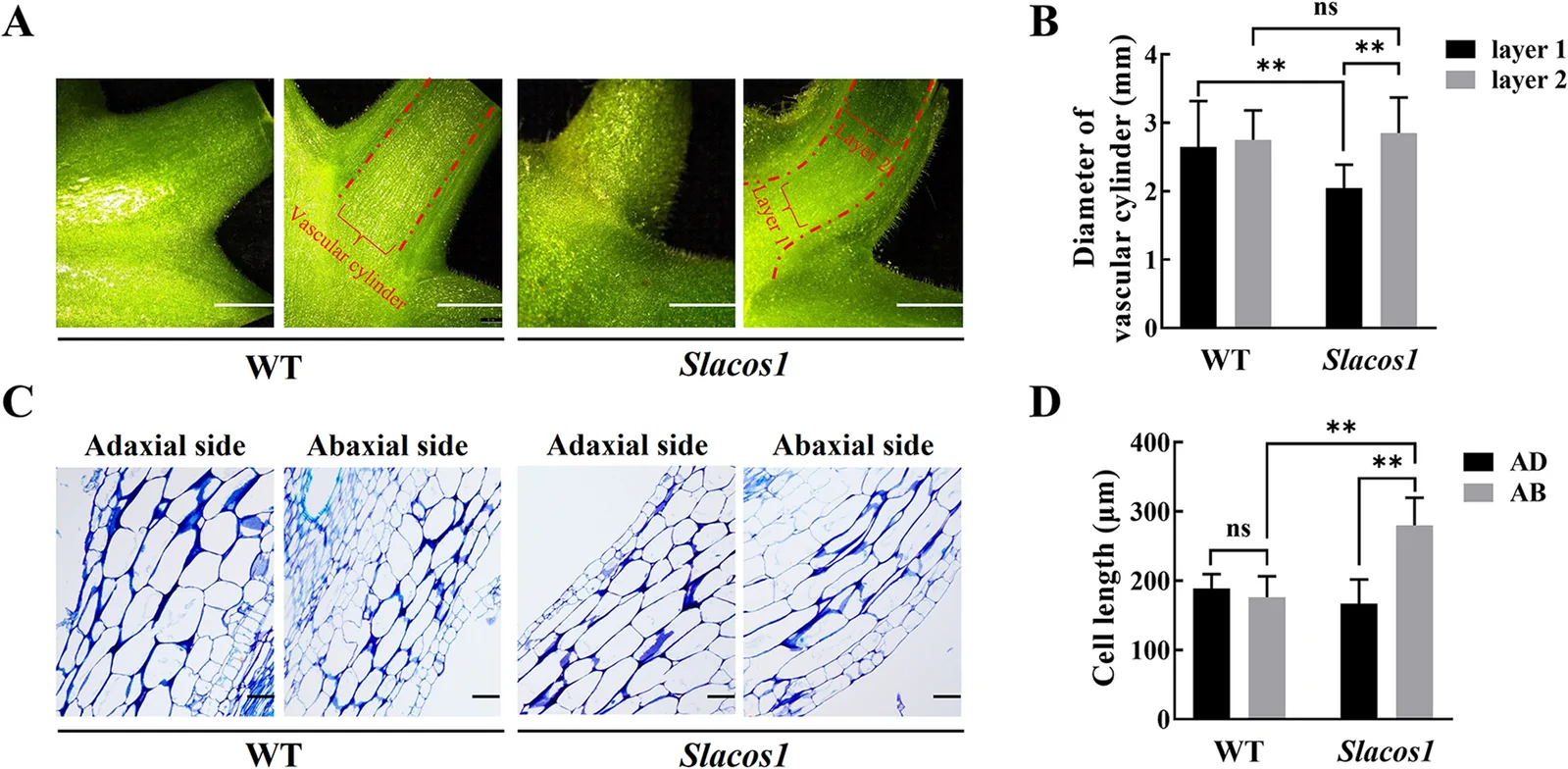
Histology analysis between *the Slacos1* mutant and WT. **A** Magnified images of the surface and longitudinal section of the branch base (about 1.5 cm segments from the joint of stem and branch) at the 4-5th node of 18-week-old plants. Bars = 3 mm; **B** The diameter of WT and *Slacos1* vascular cylinder in the branch base (*n* = 9 individual plants). The vascular cylinder and the position of layer1 and layer 2 were signed by dotted red lines; **C** Branch longitudinal sections stained with Toluidine Blue. AD denotes the half segment of the branch facing toward the main stem or the biological upper half of the branch, and AB is opposite (Fig. S1). Bars = 50 μm; **D** The length of the AD and AB cells (*n* = 9 individual plants). Values are means ± SD. Student’s *t*-test: ns, no significant; **, *P* < 0.01

In order to investigate how *SlACOS1* influenced the branch angle, we monitored the microstructure of branch using histologic sections. First of all, the magnified surface of the branch bases was observed by stereo-microscope. In WT, the branch was growth obliquely but straightly with a larger angle. Oppositely, the branch of *Slacos1* was curve upwardly, resulting in a smaller angle (Fig. 2A). A vertical section was used to divide the branch bases into two to observe the interior tissue structure (Fig. 2A). As we can see, all the branch tissue structure, from outside to inside, including epidermis, cambium, phloem, xylem and pith grew in normal direction and parallel orient along the branch, and there was little macroscopic difference between the two sides of the WT branch (Fig. 2A). And the diameters of vascular cylinder composed by cambium, phloem, xylem and pith was 2.8 mm with almost the same length in every observed position (Fig. 2B). However, the *Slacos1* branch tissue showed extremely difference with that of WT, where the vascular cylinder was upwardly curve with a changed thickness (Fig. 2A). And the smallest thickness of vascular cylinder was 1.6 mm which was significantly reduced compared to the 2.7 mm in WT (Fig. 2B). In *Slacos1*, the mean cell length, which was measured approximately in the most curve position about 3–5 mm from the branch–stem junction in the abaxial side (AB) was 280 μm and significantly (*p* < 0.01) longer than the adaxial side (AD,167 μm) and the AB (176 μm) of WT, while there was no difference (ns) between the AD (189 μm) and AB (176 μm) in WT (Fig. 2C and D).

**Fig. 3:**
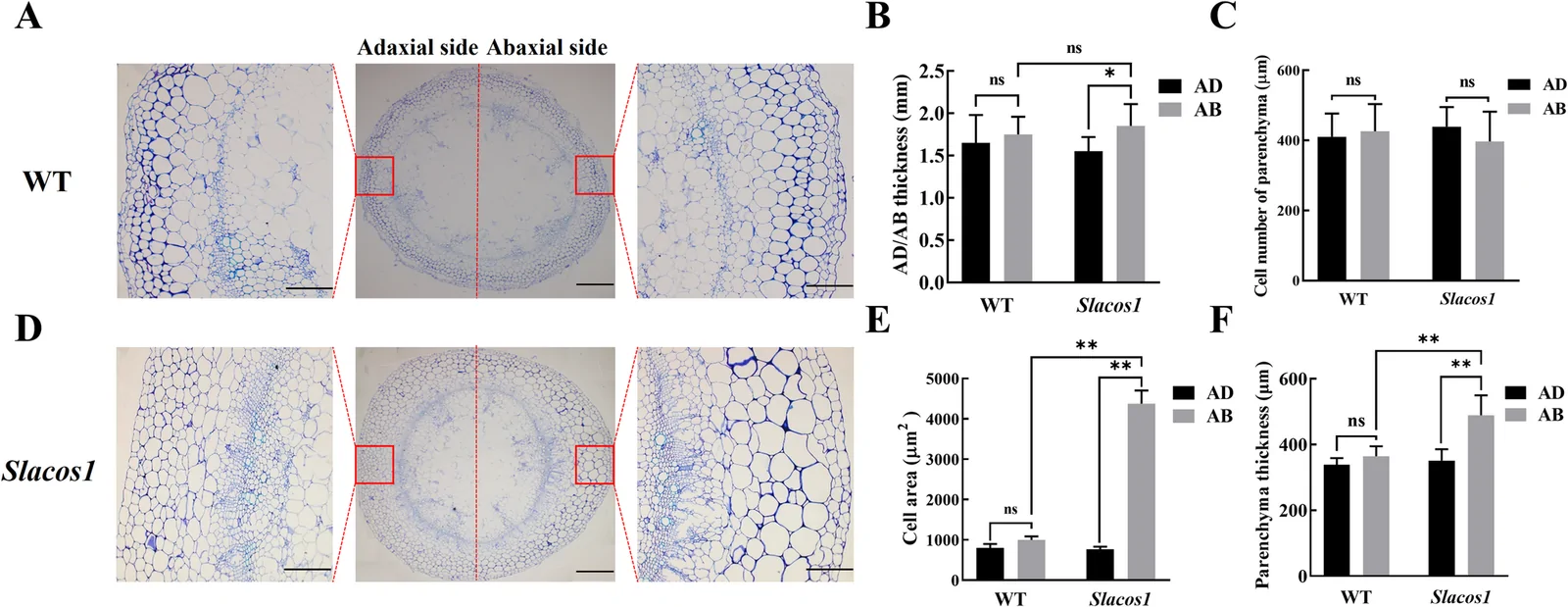
The branch cellular architecture phenotype of the WT and *Slacos1* mutant. All samples were collected from 18-week-old tomato plants. **A** and **D** Cross sections stained with Toluidine Blue. The most curved part of the branch (~ 0.5 cm, signed by red boxes in Fig. S1.) was cut and sliced. Bars = 500 μm. The left and right images showed the magnified tissue cells signed by the red lines. Bars = 100 μm; **B** The thickness of AD and AB. It represented the maximum thickness of the AD/AB from axle wire (blotted red lines) to branch edge; **C** The cell number of the parenchyma tissue in AD and AB; **E** The cell size. Only the parenchyma tissue cells were measured; **F** The thickness of branch parenchyma tissue. Parenchyma tissue was indicated in Fig. S1. Quantifications in B, C, E and F were performed using samples from 9 individual plants, with a total of 40 biological sections analyzed. Values are means ± SD. Student’s *t*-test: ns, no significant; *, *p* < 0.05, **, *P* < 0.01

In transverse sections, the AB side of the branch was elongated and thicker than AD, and the cell size was also bigger than AD in *Slacos1* branch, whereas the WT branch had no evident difference in everywhere in the branch (Fig. 3A and D). Data statistics were found that the branch transverse semidiameter of the AB in *Slacos1* (1.9 mm) was significantly (*p* < 0.05) bigger than AD (1.5 mm) with almost the same length with AD (1.7 mm) and AB (1.8 mm) in WT (Fig. 3B). The thickness of the parenchymatous tissue (from endodermis to phloem; signed in Fig. S1) in AB was 489 μm and significantly (*p* < 0.01) thicker than AD (351 μm) in *Slacos1* or AB (364 μm) and AD (338 μm) in WT (Fig. 3F). After examining parenchymatous tissue cells number and size, we confirmed that this morphology phenotype was caused by the increase of cells size instead of number. Expectedly, comparison of the parenchymatous tissue cells number and size showed no significant difference between the AD and AB in WT (Fig. 3C and E). However, the *Slacos1* cell size in AB (4376 µm^2^) was more than 4.3 times larger than AD (998 µm^2^), whereas the cells number remained unchanged (Fig. 3C and E). Those above results revealed that the curved branch in *Slacos1* was caused by the cell elongation and expansion in AB side of the branch.

### *SlACOS1* is involved in auxin signaling pathway

**Fig. 4:**
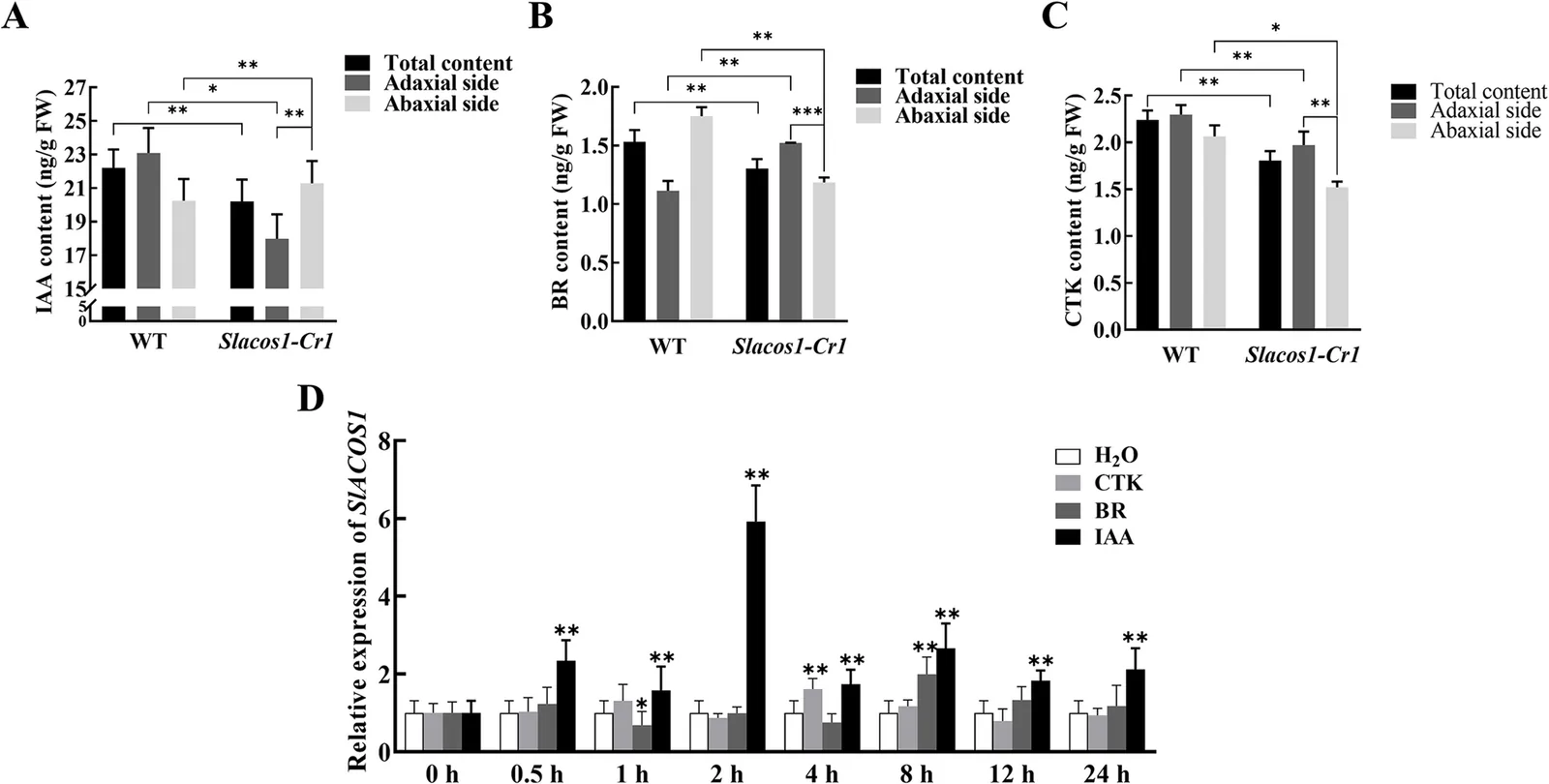
The content of several plant hormones in WT and *Slacos1* branch and the response of *SlACOS1* to phytohormones. **A** IAA (auxin); **B** BR (brassinosteroid); **C** CTK (cytokinin). Tomato plants at the nine-leaf stage were used for A-C, with 9 individual plants used for each treatment. **D** The relative expression of *SlACOS1* is detected by quantitative real-time PCR in WT seedlings. WT seedlings with 5 fully expanded true leaves were separately treated with individual phytohormones, and H₂O treatment served as the control. A total of 9 individual plants were used, with every 3 plants pooled as one biological replicate. *EF-1α* is used as reference gene. Values are means ± SD. Student’s *t*-test: *, *P* < 0.05, **, *P* < 0.01, the ns (no significant) is not showed

It has been reported that plant hormones such as auxin, cytokinin and brassinosteroid played a crucial important role in regulating branch angle [3]. Hence, we measured the content of IAA, CTK and BR in WT and *Slacos1* branch. The results showed that all the three hormones content in the whole branch was significantly reduced in *Slacos1*, 20 ng/g for IAA, 1.3 ng/g for BR and 1.8 ng/g for CTK compared to 22.2 ng/g for IAA, 1.5 ng/g for BR and 2.3 ng/g for CTK in WT (Fig. 4A, B and C). In WT, the IAA content was higher in AD (23.08 ng/g) than AB (20.25 ng/g), while the content in AD decreased to 17.98 ng/g and was significantly lower than AD (21.29 ng/g) in *Slacos1* (Fig. 4A). The BR and CTK content were significantly higher in AD than AB whatever in WT or *Slacos1* with the same trend (Fig. 4B and C). Moreover, in AD samples, BR and CTK content in WT were also higher than in *Slacos1* with no difference in AB (Fig. 4B and C). The results suggested that mutation of *SlACOS1* disrupted the original content of hormones, especially auxin and resulted in a new asymmetric distribution in branch to regulate the asymmetric growth.

To further study the relationship between *SlACOS1* and those hormones, we determined whether IAA, CTK and BR could induce the expression of *SlACOS1*. The WT plants were treated by exogenous IAA, CTK and BR hormones for 0, 0.5, 1, 2, 4, 8, 12 and 24 h to detect the transcript levels of *SlACOS1*. The RT-qPCR results showed that *SlACOS1* expressed significantly increased (2.4-fold) after only 0.5 h of IAA treatment compared to 0 h (Fig. 4D). The peak expression level was obtained after 2 h treatment, with approximately 6 times higher than control. Afterward, its expression gradually decreased with time extension (Fig. 4D). On the whole, the expression of *SlACOS1* increased at first and then decreased after treatment by IAA. However, BR and CTK treatment only resulted in a slightly difference at 1, 4 and 8 h which meant that those two exogenous hormones cannot induced the expression of *SlACOS1* (Fig. 4D). The results revealed that *SlACOS1* only responded to IAA instead of BR and CTK.

**Fig. 5:**
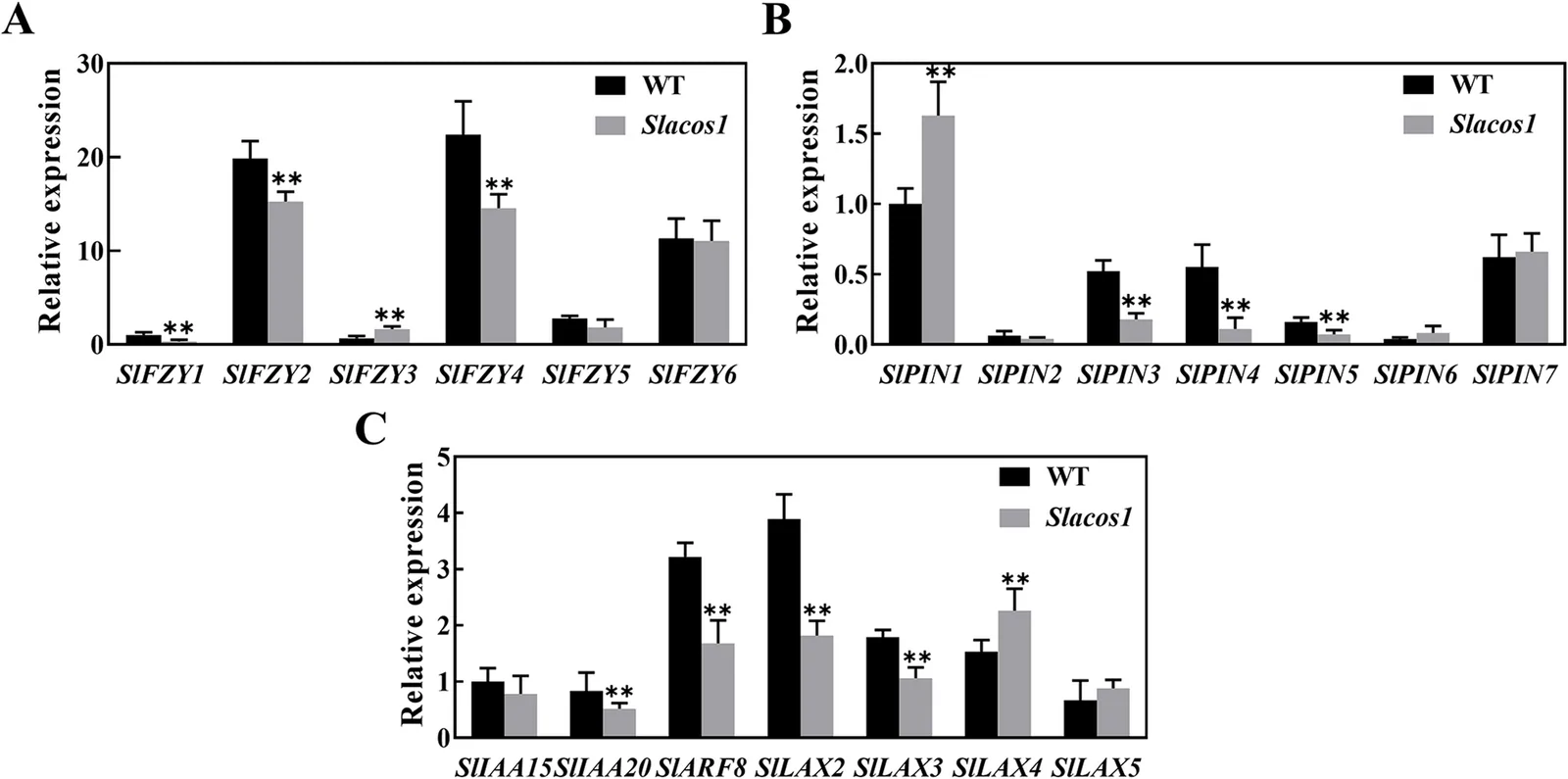
Knockout of *SlACOS1* impacted the expression of a series of IAA signaling pathway genes. **A** The relative expression of tomato auxin biosynthesis genes *SlFZYs*; **B** The relative expression of the auxin efflux-facilitating *PIN-FORMED* (*SlPIN*s); **C** The relative expression of like auxin influx carrier (*SlLAXs*) and *SlIAA15*, *SlIAA20* and *SlARF8*. A total of 9 individual plants at 18-week-old were harvested, with every 3 plants pooled as one biological sample. *EF-1α* was used as reference gene. Values are means ± SD. Student’s *t*-test: **, *P* < 0.01, the ns (no significant) is not showed

Based on the above results of the altering auxin content and the sensitivity of *SlACOS1* to auxin, we detected the expression of auxin biosynthesis, polar transport and response-related core genes to verify the effect of *SlACOS1* to auxin. The RT-qPCR results showed that a number of IAA signaling pathway genes were suppressed in *Slacos1* mutants. Among them, the core components of IAA biosynthesis *SlFZYs* genes, including *SlFZY1*, *SlFZY2* and *SlFZY4*, were significantly suppressed in *Slacos1* (Fig. 5A). The expression of several *SlPINs* (*SlPIN3*,* SlPIN4* and *SlPIN5*), the key genes of polar auxin transport, were also strongly inhibited in *Slacos1*. Meanwhile, the expression of *SlPIN1* was upregulated for more than 1.6 times compared to WT (Fig. 5B). Furthermore, the IAA transcriptional regulator gene *SlIAA20* was detected with significantly decreased expression in *Slacos1*. An auxin influx carrier-like gene *SlLAX4* expressed with about 1.5-fold more abundant than WT with significantly downregulation of *SlLAX2/3*. What’s more, we also found the transcripts of an auxin response factor *SlARF8* accumulated more than 2-fold lower than WT (Fig. 5C). Other genes (*SlFZY5*/*6*, *SlPIN2/6/7*, *SlIAA15* and *SlLAX5*) detected by RT-qPCR did not present significant difference between WT and *Slacos1* (Fig. 5A, B and C). The results showed that editing *SlACOS1* influenced the expression of IAA-related genes, and the changes in gene expression partly explained the asymmetric auxin distribution.

### *SlACOS1* is involved in lignin pathway by interacting with *SlCCR5* and *6*

**Fig. 6:**
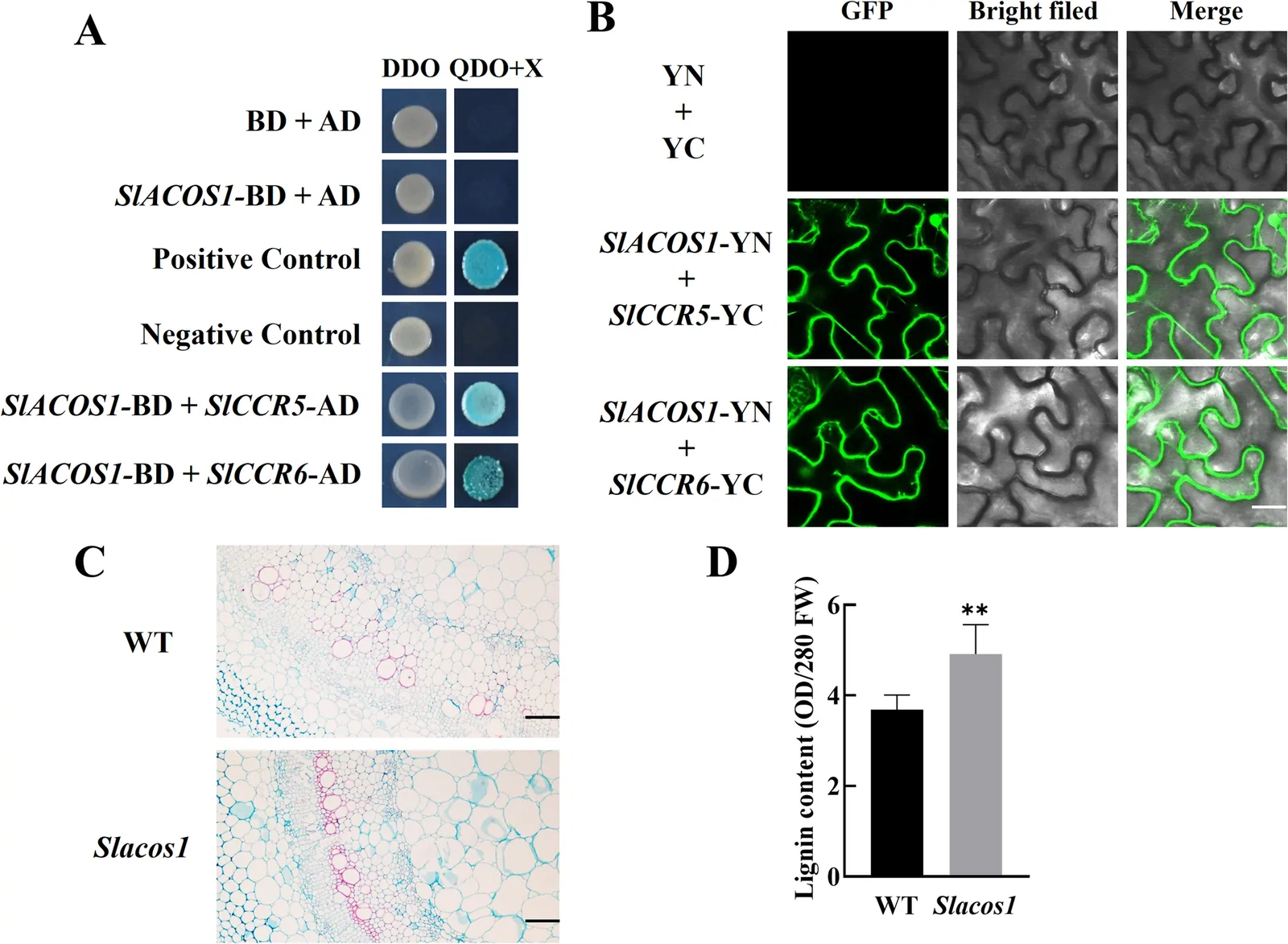
*SlACOS1* interacted with *SlCCR5/6* and regulates the lignin content. **A** Yeast two-hybrid analysis. AD + BD was used as empty vector control. *SlACOS1*-BD + AD was used to detect the self-activation. pGBKT7-Lam + pGADT7-T was used as negative control. pGBKT7-53 + pGADT7-T was used as positive controls. DDO was the SD/-Trp/-Leu yeast culture-medium, and QDO + X was the SD/-Trp/-Leu/-Ade/-His/ x-α-Gal medium; **B** BiFC analysis in tobacco epidermal cells. Empty vectors (YN + YC) were used as negative controls. Bar = 50 μm; **C** Phloroglucinol staining for lignin content in the WT and *Slacos1* branch. Bar = 100 μm; **D** The lignin content in the WT and *Slacos1* branch. Values are means ± SD (*n* = 3). Student’s *t*-test: **, *P* < 0.01

In order to detect the signaling pathway that *SlACOS1* participated in, *SlACOS1*-interacting proteins were screened by yeast two-hybrid cDNA library of tomato. Two SlCCR proteins encoded by *Solyc03g097170* and *Solyc08g076790* were identified to interact with SlACOS1 (Fig. 6A). *CCR* gene encoded cinnamoyl-CoA reductase which was the first committed enzyme of the lignin-specific branch in the phenylpropanoid metabolic network. Phylogenetic analysis showed that those two *SlCCRs* were highly homology with alfalfa (*Medicago truncatula*) *MtCCR1-5/6* and formed a distinct clade (Fig. S3). According to the homologous *MtCCR1-5* and *6*, *Solyc08g076790* and *Solyc03g097170* were respectively named as *SlCCR5* and *SlCCR6* (Fig. S3). A bimolecular fluorescence complementation (BiFC) analysis was performed to further confirm the interaction between *SlACOS1* and *SlCCR5/6* in tobacco (*Nicotiana benthamiana*) leaf cells. When *SlACOS1*-nYFP and *SlCCR5/6*-cYFP were transiently co-expressed, green fluorescence was observed in the plasma membrane, revealing the interaction of *SlACOS1* and *SlCCR5/6* in plants (Fig. 6B). It has been reported that CCRs were lignin synthase enzymes and controlled the lignin biosynthesis [35] and lignin regulated the branch angle [36]. Thus, we detected the lignin content in WT and *Slacos1* to determine whether the alter branch angle in *Slacos1* was related to lignin. The lignin content in *Slacos1* branch was about 4.91 (OD/280 FW) which was significantly higher than 3.68 (OD/280 FW) in WT branch (Fig. 6D). Lignin staining revealed that vascular bundles and mechanical tissues in the internode of *Slacos1* had darker red color and larger stained region than WT (Fig. 6C). It meant that *Slacos1* had higher lignin content and was intensely lignified, whereas WT was relatively weakly lignified. Taken together, these results demonstrated that the change of *Slacos1* branch angle was related to the increased content of lignin, and *SlACOS1* regulated the lignin biosynthesis by interacting with two lignin synthase enzymes *SlCCR5/6*.

### *SlACOS1* is involved in gravitropic response but not gravity perception

**Fig. 7:**
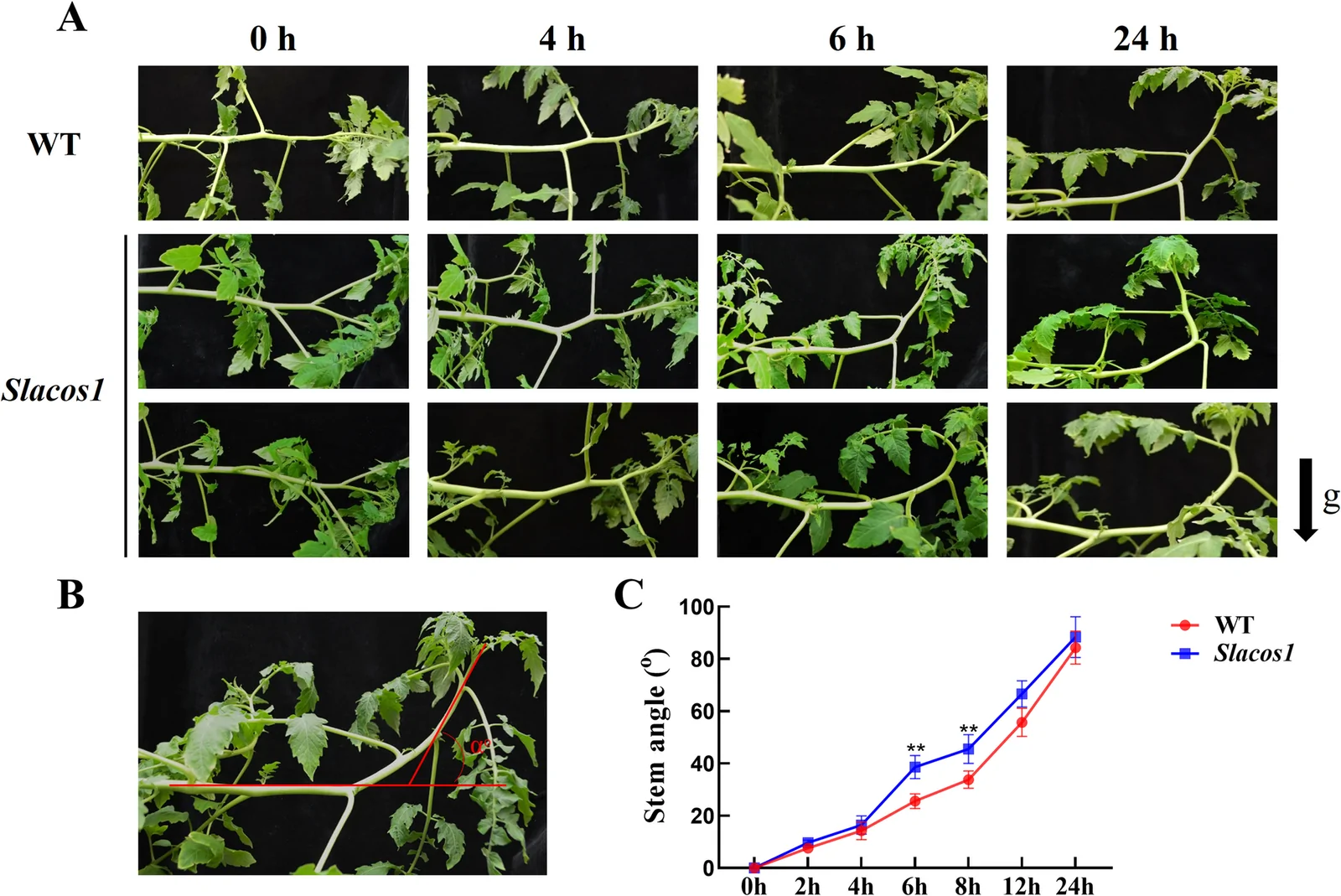
Gravitropism analysis of the WT and *Slacos1*. **A** The growth situations of WT and *Slacos1* plants in respond to gravity for 0, 4, 6 and 24 h. **B** The α indicated with red lines represents the stem bending angle. **C** The statistics of the degree of bending of plant stems placed horizontally at 0, 2, 4, 6, 8, 12 and 24 h. Values are means ± SD (*n* = 12). Student’s t-test: **, *P* < 0.01

Previous studies have suggested that shoot gravitropism contributes greatly to the control of branch or tiller angle [4, 37]. To explore whether *SlACOS1* was involved in the perception of gravity, we horizontally placed WT and *Slacos1* plants to 90° inverted gravity processing to observe the degree of stem bending (Fig. 7A). After 0, 2, 4, 6, 8 12 and 24 h of inversion, the angle of the stem deviating from horizontal direction was recorded by camera and measured (Fig. 7A). The *Slacos1* plants clearly bent upward slightly at 4 h, while no visible bending was observed in the WT plants. After 6 h treatment, a significant difference in bending angle was observed between the *Slacos1* and WT plants. After 8 h of inversion, the bending angles reached maximum difference between WT (34°) and *Slacos1* (54°). In addition, the bending angle differences in WT and *Slacos1* were dying out gradually after 12 h (Fig. 7C). The results indicated that *SlACOS1* may play a role in the response to gravitropism in tomato plants.

**Fig. 8:**
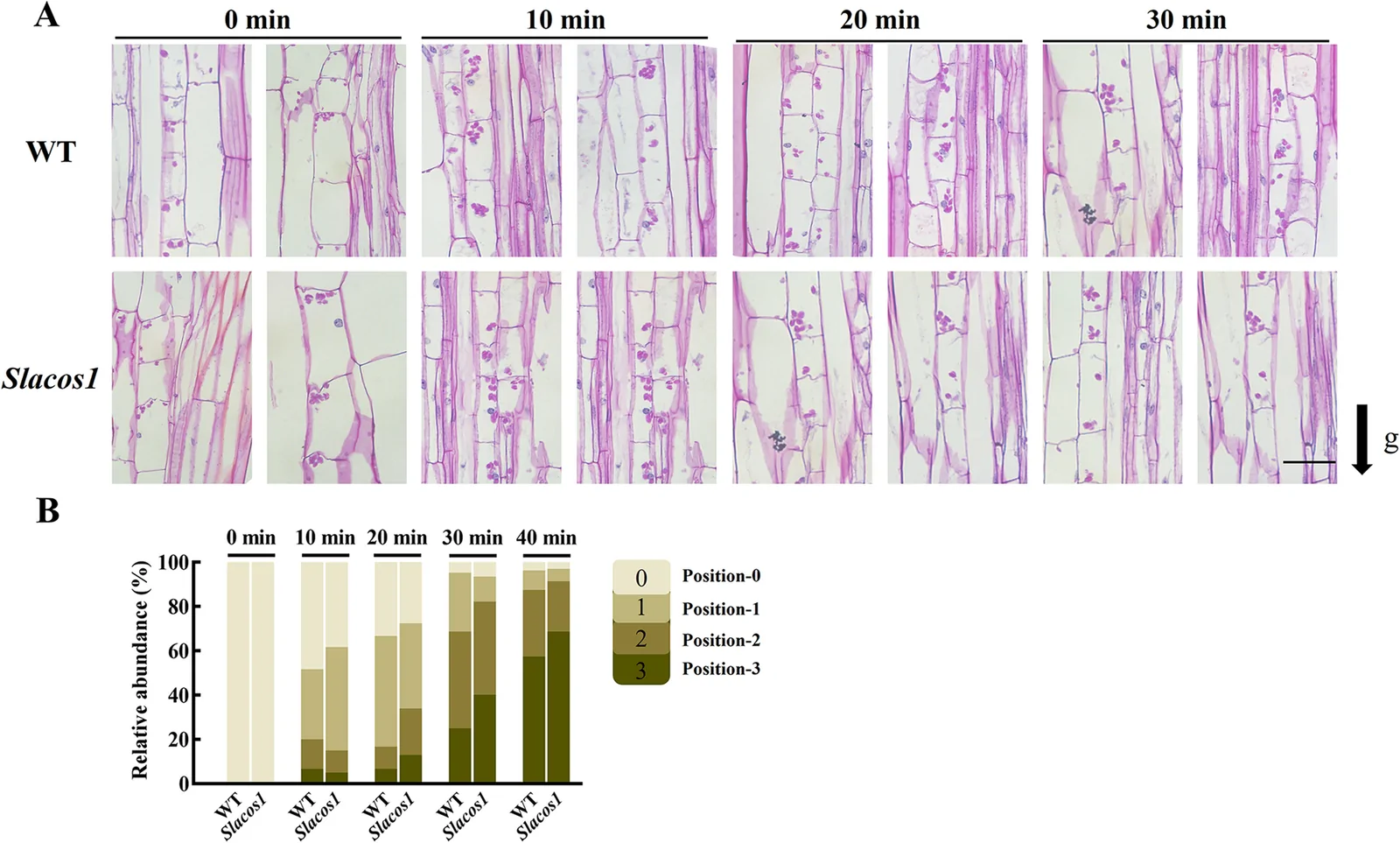
The sedimentation of amyloplasts of the WT and *Slacos1.* **A** Kinetic comparison of the sedimentation of amyloplasts along the direction of gravity in parenchyma cells between the wild WT and *Slacos1*. The red dots in cells are amyloplasts staining by Periodic acid-Schiff. Bar = 50 μm. **B** The relative number of amyloplasts situated in different position of the parenchyma cells at different time points. The schematic cell was used to determine the position of amyloplasts. Each cell was divided into four equal segments along its length, numbered position 0 to 3. When stem is inverted, amyloplasts move along the basal-apical axis and their positions are scored. The arrow indicates the direction of gravity (g)

To confirm the role of *SlACOS1* in gravitropism, Periodic Acid-Schiff (PAS) staining was used to detect amyloplasts in statolith cells and examine the starch–statolith hypothesis in *Slacos1* mutants [38]. Amyloplasts normally localize at the bottom of statoliths and sediment toward the morphological upper end when plants are inverted (Fig. 8A). We observed amyloplast sedimentation in the basal region of branches after whole-plant inversion. Each cell was divided into four equal parts (positions 0 to 3 from top to bottom), and amyloplast distribution was quantified at different time points. At 0 min, PAS-stained amyloplasts were exclusively located at position 0 in both WT and *Slacos1*. Under gravity stimulation, amyloplasts gradually sedimented toward position 3. At 10 min, the proportions of amyloplasts at positions 0–3 were 38.33%, 46.67%, 10.00%, and 5.00% in *Slacos1*, versus 48.33%, 31.67%, 13.33%, and 6.67% in WT, with no significant difference (Fig. 8B). Similarly, no significant differences were observed at 20, 30, or 40 min. These results indicate that *SlACOS1* does not affect gravity perception or amyloplast sedimentation in tomato. Although *Slacos1* plants bent more rapidly than WT in the gravitropism assay, it may be caused by the higher degree of lignification of to facilitate plants upward growth.

## Discussion

Branch angle shapes plant architecture into a loosen or dense structure, and thus influences the light interception and competition between neighboring plants in natural settings, ultimately contributing to the plant yield and productivity [2]. Due to its important contribution to agronomic trait, lots of genes controlling branch or tiller angle have been identified in different plants [11]. For example, *Tiller Angle Control* is one of major controlling genes, and loss-of-function in plants contribute to erect and compact growth. In *Arabidopsis*, *tac1* mutants show significantly narrower lateral branch angles than the wild-type [39], and *Ppetac1* mutants “*br*” also show small branch angle and have a more erect plant architecture in peach (*Prunus persica*) [40]. In this study, *SlACOS1* gene was knockout by CRISPR/Cas9, and the *Slacos1* mutant demonstrated an evident erect growth phenotype with small branch angle in tomato (Fig. 1). The *Slacos1* phenotype of branch angle was consistent with those *tac1* mutants in diverse plant species. However, Y2H assays showed no direct interaction relationship between *SlACOS1* and *SlTAC1* (Fig. S4). Those results indicated that *SlACOS1* performed function in setting the orientation of lateral branches, but may be independent on *TAC1*.

Plant development is determined by a combination of three major cell biological processes: cell division (proliferation), differentiation and growth (expansion and elongation) [41]. A notable example is hypocotyl elongation and apical hook formation caused by cell elongation which is driven by asymmetric auxin distribution across the hypocotyl [42]. The rice *Loose Plant Architecture 1* mutant *lpa1* shows larger tiller angle and leaf angle due to faster and enhancer elongation on the adaxial side of the lamina joint [6]. The *Slacos1* branch grew bending upward caused by the increased parenchymatous tissue elongation on the abaxial side (AB) of branch base with larger cell length (Fig. 2C and D). Moreover, scanning the transverse sections of *Slacos1* branch base was found that the cell size was significantly larger on AB than AD and both sides of WT with little change in cell number (Fig. 3A, D and E). The cell expansion resulted in the unequal growth rapid on the two sides of *Slacos1* branch and a more thickness semidiameter of AB than AD (Fig. 3), which contributed to the branch bent upward.

Almost all the process of plant growth and development are controlled by the plant hormones. In sweet oranges (*Citrus sinensis*), *CsTAC1* regulates the branch angle by modulating the endogenous hormone content with lower auxin contents and increased cytokinin levels [43]. However, in this study, auxin, cytokinin and brassinosteroid contents were all reduced in the whole branch of *Slacos1* mutant lines (Fig. 4A, B and C), disagreed with the results of Dutt et al. [43]. BR related mutants, such as *dwarf61* (*d61*), *Oslic*, *Osdwarf4–1* and *m107* (a gain-of-function mutant in BR biosynthetic gene *D11*), showed altered tiller or leaf orientation by disrupting the BR biosynthesis or signaling [17, 18, 44, 45], which were partly similar to the upward growth of branch with the decrease of BR content in *Slacos1* (Fig. 4B). Especially for auxin, the branch angle is regulated by not only the relative auxin content but also the asymmetric distribution. The rice *lazy1* (*la1*) mutant, which shows wide-angled branch phenotype, reveals that *LA1* gene acts on the polar auxin transport stream to redistribute auxin preferentially to the lower side of the shoot. Consistently, the unequal distribution of auxin was detected with an increase of relative content on AB of the branch than AD in *Slacos1* compared with WT (Fig. 4A). It meant that knockout *SlACOS1* altered the redistribution of auxin in branch, where it triggered growth in AB and in turn led to bending.

Auxin distribution patterns and polar transport are directly driven by different auxin transmembrane carriers, including the auxin influx carrier (AUX1)/LIKE AUX1 (LAX1) protein family [46], the auxin efflux-facilitating PIN-FORMED (PIN) family [47] and a number of the p-glycoprotein ABC transporters [48]. This progress is also involved in a trans-organ differential in the degradation of *AUX/IAA* proteins by the auxin receptor-E3 ubiquitin ligases, and subsequent gene expression changes promoted by *ARF* proteins such as *NPH4/ARF7* [49, 50]. For instance, rice *LA2* regulates shoot gravitropism and tiller angle through the asymmetric expression of *OsIAA20* and auxin redistribution [37]. In *Slacos1*, most *SlPINs* and *SlAUXs* were down-regulated, whereas other genes such as *SlPIN1* and *SlLAX4* showed higher expression (Fig. 5A-C). The results could partly interpret the redistribution of auxin in *Slacos1* branch. These apparently “contradictory” gene expression patterns may be caused by conditional gene function, exactly as over-expression of *OsPIN2* enhances auxin transport and leads to increased rice tiller angle [51], while the *OsPIN1b* RNAi mutant plants show a larger tiller angle [52]. Thus, disrupted expression of auxin biosynthetic and transport genes contributes to the exaggerated asymmetric auxin gradient in *Slacos1*, though the molecular intermediates connecting SlACOS1 acyl-CoA metabolism to these transcriptional shifts remain unclear, and only a correlative relationship can be established at present, which demands further research.

According to the Cholodny-Went hypothesis, gravity-induced asymmetric auxin distribution results in differential cell elongation and organ bending in higher plants [53]. A number of studies have been verified that the theory applied to the regulation mechanisms of tiller or branch angle. Gravitropism analysis showed that *Slacos1* plant stems bent upward more quickly than WT guided by gravity (Fig. 7). Amyloplast sedimentation is believed to be a primary and symbolic event in gravity sensing and auxin redistribution [54]. However, the amyloplast sedimentation rate showed no significant difference between *Slacos1* and WT in statolith cells (Fig. 8), indicating that gravity perception by amyloplast sedimentation is not affecte in *Slacos1*. But asymmetric auxin redistribution was still observed during the bending process (Fig. 7). These observations suggest that *SlACOS1* does not function in the gravity perception or early auxin redistribution steps, but rather acts downstream in a process that might modulate the efficiency of the bending response.

Lignin is a major structural component of the cell walls that provides mechanical strength for the vascular systems thereby facilitating plants upward growth [55–58]. In bermudagrass (*Cynodon dactylon* L.), the tiller angle and stem growth direction are changed by lignin in erect-growing lines [36]. We also found the lignin content of *Slacos1* was higher than WT (Fig. 6C and D), which might support the erect growth of branch. Acetyl-CoA has been identified as the donor substrate to feruloyl-CoA monolignol transferases ρHBMTs that acylate monolignols [59], suggesting that *acetyl-CoA* biosynthesis may directly participate in the biosynthesis pathway of lignin. *SlACOS1* could interact with two tomato *cinnamoyl-CoA reductase* genes, *SlCCR5/6* confirmed by Y2H and BiFC (Fig. 6A and B), and the total lignin content was increased in *Slacos1* branch (Fig. 6C and D). Furthermore, in tomato (*Solanum lycopersicum* L. cv. Microtom), two CCR genes (*LeCCR1* and *2*), are identified and characterized to regulate lignin biosynthesis, and reduced *LeCCR1* expression leads to growth defect, leaf morphological changes, and decreased lignin content [60]. In addition, the *LeCCR1* greatly influences the growth of vascular bundles and the xylem ring with a partly similar function to *SlACOS1* (Figs. 2 and 3). Thus, we speculated that the *Slacos1* could influence the lignin biosynthesis to supply more lignin for the formation of secondary cell walls in the progress of cell elongation and expansion. Notably, the faster upward bending of *Slacos1* is not purely mechanical nor a simple hyper-gravity response (Figs. 7 and 8). Auxin redistribution sets the direction and triggers differential cell elongation, while elevated lignin strengthens cell walls and reduces tissue resistance, thereby amplifying the bending magnitude induced by the same auxin gradient. Auxin might determine the growth asymmetry, whereas lignification modulates the final bending speed and angle. *SlCCR5* and *6* were considered as the putative genes in the signaling pathway of *SlACOS1* regulating branch angle. However, additional experiments need to be performed to prove this speculation.

## Conclusions

In this study, we identified and characterized a tomato *Acetyl-CoA synthetase* gene named *SlACOS1* which was relative to regulate the branch angle. Editing *SlACOS1* using CRSPR/Cas9 resulted in smaller branch angle in tomato line PI 114,490. *Slacos1* mutants disrupted the asymmetrical auxin distribution to enhance the cell elongation and expansion and the asymmetric growth in the abaxial side (AB) of the branch. *SlACOS1* interacted with two lignin biosynthesis gene *SlCCR5/6* and altered the lignin content and the degree of lignification of the branch to support the branch bending. Taken together, *SlACOS1* regulated branch angle by modulating the asymmetric distribution of auxin and lignin biosynthesis in tomato.

## Supplementary Information

Supplementary Material 1.

## Data Availability

All data generated or analysed during this study are included in this published article and its supplementary information files.

## Abbreviations

AD: Adaxial Side
AB: Abaxial Side
IAA: Auxin
CTK: Cytokinin
BR: Brassinosteroid
BiFC: Bimolecular Fluorescence Complementation
Y2H: Yeast two-Hybrid
CCR: Cinnamoyl-CoA Reductase
