# Supplementary material for: Functional characterization of Acyl-CoA Synthase 1 in tomato branch angle development
Source: BMC Plant Biol. 2026 Jul 14;26:1716. doi: 10.1186/s12870-026-09516-4 (PMC13647794; doi:10.1186/s12870-026-09516-4)
Supplement: Supplementary file 1 [file 12870_2026_9516_MOESM1_ESM.docx]

**Functional characterization of *Acyl-CoA Synthase 1* in tomato branch angle development**

Mengyi Yu^1,2,3*^, Zhihuan Ge^3,4*^, Yinge Xie^3,4#^, Yongjie Qi^1,3#^

*^1^Key Laboratory of Horticultural Crop Germplasm Innovation and Utilization (Co-Construction by Ministry and Province), Institute of Horticulture, Anhui Academy of Agricultural Sciences, Hefei 230001, China*

*^2^Beijing Key Laboratory of Growth and Developmental Regulation for Protected Vegetable Crops, Department of Vegetable Science, China Agricultural University, Beijing 100193, China*

*^3^Anhui Provincial Key Laboratory for Germplasm Resources Creation and High-Efficiency Cultivation of Horticultural Crops, Hefei 230031, China*

*^4^Institute of Vegetables, Anhui Academy of Agricultural Sciences, Hefei 230001, China*

late lignin biosynthesis, affecting branch angle independently of gravity perception.

^#^Corresponding author: Yinge Xie; Yongjie Qi

e-mail: 552126921@qq.com; anhuiqyj@163.com

*These authors have contributed equally to this work.

**Table S1.** The primer sequences used in this study.

**Figure S1.** Schematic diagrams.

**A** The dashed boxes indicate the position of sampling for histologic sections and staining; **B** The red arrows point at the adaxial side (AD) and abaxial side (AB) of the branch; **C** The position of parenchyma tissue is signed by red line. All samples were collected from 18-week-old tomato plants.

**Figure S2.** Tissue-specific transcript accumulation patterns of *SlACOS1.*

RT-qPCR is used to detected the mRNA accumulation of *SlACOS1* gene in different tissues and organs in tomato line PI 114490 at 18 weeks. *EF-1α* (*Solyc06g005060*) is used as the internal control gene. Rt: root; St: stem; Ab: axillary bud; Yb: young branch; Mb: mature branch; Yl: young leaf; Ml: mature leaf; Fl: flower; Mg: mature green fruit; Rf: ripening fruit. The experiments are carried out with three biological replicates and three technical replicates. Error bars represent the SD. Significant differences were analyzed by one-way ANOVA, and different lowercase letters denote significant differences at P < 0.05.

**Figure S3.** Phylogenetic analysis of *CCR* genes family.

The full-length amino acid sequences of CCR proteins from tomato (*Solanum lycopersicum*; LeCRR1/2 and SlCCR5/6), *Arabidopsis* (*Arabidopsis thaliana*; AtCCR1/2 and AtCCR-like1/2/3/4), poplar (*Populus trichocarpa*; PtrCCR1/2/3/4/5/6/7/8/9/10/11) and alfalfa (*Medicago truncatula*, MtCCR1-1/2/3/4/5/6) were aligned by ClustalX (http://www.clustal.org/), and the phylogenetic tree was constructed using the neighbor-joining method with 1000 bootstrap replicates by MEGA 5.0 (<https://megasoftware.net/>).

**Figure S4.** The interaction relationship between *SlACOS1* and *SlTAC1*.

Yeast two-hybrid analysis shows that *SlACOS1* cannot interact with *SlTAC1* directly. AD + BD were used as empty vector control. *SlACOS1*-BD + AD was used to detect the self-activation. pGBKT7-Lam + pGADT7-T was used as negative control. pGBKT7-53 + pGADT7-T was used as positive controls. DDO was the SD/-Trp/-Leu yeast culture-medium, and QDO + X is the SD/-Trp/-Leu/-Ade/-His/ x-α-Gal medium.
